# Undergraduate Public Health Education: Does it Meet Public Health Workforce Needs?

**DOI:** 10.3389/fpubh.2015.00001

**Published:** 2015-01-26

**Authors:** James W. Holsinger, Andrea L. Lewis, Quan Chen

**Affiliations:** ^1^Department of Preventive Medicine, University of Kentucky, Lexington, KY, USA; ^2^Department of Health Management and Policy, University of Kentucky, Lexington, KY, USA

**Keywords:** undergraduate, bachelor’s degree, professional degrees, academic degrees, public health practitioner

## Introduction

The professional bachelor’s degree [Bachelor of Public Health (BPH) or Bachelor of Science (BS) in Public Health] provides a cadre of trained individuals to fill entry-level positions in American public health agencies. The traditional Bachelors of Arts or Science degrees with majors in public health produce an informed citizenry, but may not provide sufficient public health course content to enable graduates of such programs to effectively enter the public health workforce.

## Background

The 2003 report of the Institute of Medicine (IOM) Committee on Educating Public Health Professionals for the twenty-first century anticipated that developing broad undergraduate public health education would result in a cadre of trained individuals to enter the public health workforce as well as provide a “public health perspective” to their worksite (1). The report also called training in public health an essential part of the education of an informed citizenry. These concepts have been the underlying themes on which undergraduate public health education has developed over the last decade. As a result, Riegelman and Albertine have stated: “undergraduate public health at 4-year institutions: it is here to stay” (2).

The need for additional members of the public health workforce at all levels is expected to reach 250,000 by 2020 (3, 4). These estimates, made in 2008, have been confounded by the recession of 2008–2010 and by a projected retirement eligibility rate of 29% among public health practitioners (5). To meet the required workforce projections, the graduation rate from Association of Schools and Programs of Public Health (ASPPH) schools needs to triple (3, 4). Arnold and Schneider have postulated that public health undergraduates completing their degrees and entering the workforce have the potential of relieving the pressure on graduate programs by providing entry-level practitioners (6).

Although bachelor’s degree prepared individuals may help to reduce the projected shortage of public health practitioners, the variety of bachelor’s degree programs and their variability may impede these efforts. A pilot review of programs at colleges and universities with established schools of public health was conducted to determine whether there were significant differences between bachelor’s degree programs at these institutions. It should be noted that a number of the new undergraduate programs are being developed in universities without schools of public health, including liberal arts and community colleges, and such institutions may not possess the breadth and depth of faculty expertise to produce entry-level public health practitioners, although they may certainly produce graduates that meet the recommendation for producing citizens well informed concerning public health issues. As a consequence, some institutions will produce the “educated citizen” (academic), while others will educate practitioners for the public health “workforce” (professional).

Undergraduate degree programs with public health majors are based on the various public health core disciplines (administration, epidemiology, biostatistics, environmental health, and health behavior) as well as general public health. These academic majors may occur as either BA or BS degrees. The BPH degree is a professional degree functioning at the undergraduate equivalent of the Masters of Public Health (MPH). The Bachelor of Science in Public Health (BSPH), although technically an academic degree, functions as the undergraduate equivalent of the Master of Science in Public Health (MSPH).

## Review of Bachelor Degrees in Top Ranked Public Health Programs

In an effort to gain a better understanding of the undergraduate public health degrees currently being offered at colleges and universities with established public health programs, a general, preliminary review of the course content of university-based undergraduate public health degree programs was conducted. Owing to its ready availability, a convenience sample from the U.S. News top 25 schools of public health was utilized as the study set (7). A total of 28 institutions’ websites (3 institutions tied for the 25th place) were searched for undergraduate degree curricula containing public health content.

Of the 28 schools reviewed, 17 (60.7%) offered undergraduate public health programs, with 2 institutions offering public health majors in both BA and BS degree programs. Two institutions were not considered due to offering combined undergraduate and graduate programs. A total of 19 programs from 17 schools were considered, and 3 groups of degree programs emerged: public health majors in BA degree programs, public health majors in BS degree programs, and a combination of the 5 BSPH programs and the single BPH program, the latter 2 functioning as professional degree programs. The curriculum requirements listed on the websites for each of the 19 programs were reviewed by at least two authors to determine public health content. Public health content courses included general public health courses, courses in any of the five public health core disciplines, public health practicum requirements, student research projects, and any other courses considered by the program to contain public health content.

This review demonstrated that the six BA degree programs contained an average total of 120 hours of coursework with public health content consisting of an average of 34.50 semester hours or 28.8% of the total degree hours. For the majors in the seven BS degree programs, the average total degree program hours was 120.86 with public health content representing an average of 35.86 semester hours or 29.7% of the total hours. The BPH/BSPH degree programs averaged a total of 120 semester hours with 51.83 semester hours of public health content representing 43.2% of the total. Thus, the BPH/BSPH programs contained a statistically significant 47% more public health content than BA and BS degree programs with public health majors (*p* < 0.0001) (see Table 1).

**Table 1:** **Public health content hours by degree type**.

Degree type	Number of schools	Public health content hours				Mean of total credit hours (SD)	Percentage of public health content hours (%)	Comparison of mean public health content hours to BPH/BSPH	
		Mean (SD)	25th percentile	Median	75th percentile			*t*	*p*-Value
BA	6	34.50 (6.41)	30	35	36	120 (0)	28.80	−5.10	0.0005
BS	7	35.86 (7.95)	30	33	42	120.86 (22.7)	29.70	−4.18	0.0015
BA/BS	13	35.32 (7.01)	30	35	36	120.46 (16.6)	29.30	−5.13	<0.0001
BSPH/BPH	6	51.83 (5.31)	48	49	57	120 (0)	43.20	–	–

This review of bachelor degree programs in public health provides a preliminary picture of the variability between these programs. However, this is not meant to be a comprehensive analysis of these programs or their course content, but rather it should be considered a starting point for future study. A limitation of this analysis includes the number of public health specific credit hours being deduced from reviewing online bachelor program curricula. In undergraduate education, some freedom is given to students to choose additional courses as electives. Some students obtaining BA, BS, BPH, or BSPH degrees could choose to focus their additional electives on public health courses. Thus, the calculation of credit hours here only reflects what is minimally required of students to complete the degree, but the actual exposure of students to public health courses may be higher. Similarly, undergraduate students may have other opportunities to gain experience in the field of public health during their education such as working with a faculty member in the school of public health on research or a special project or participating in voluntary internships or shadowing opportunities.

In order to fully evaluate the current state of undergraduate education in public health, bachelor degree programs from accredited schools of public health, accredited programs of public health, and colleges and universities without accredited schools or programs of public health should be evaluated. Further, a more detailed analysis of course content, the public health competencies addressed, and learning outcomes of the curricula need to be considered.

## Discussion

In order to effectively fill the projected vacancies in the public health workforce, adequately prepared practitioners will be required. Undergraduate public health degree programs may be a solution to this problem. However, if graduates of such programs are to be capable of filling entry-level public health practitioner positions previously filled by individuals with an MPH degree, a level of academic content such as is found in graduate degree programs will be required. Currently, the Council on Education in Public Health (CEPH) requires that accredited MPH programs consist of at least 42 credit hours. As shown in this preliminary review, the average number of public health content credit hours in BA and BS programs fall below the MPH requirement, while the BPH and BSPH programs on average meet it. Unlike BA and BS programs, the BPH and BSPH programs mirror the content found in graduate MPH and MSPH degree programs, but at an undergraduate level.

Using the current credit hour requirements of an MPH degree as a benchmark, a BA or BS degree with a public health major may provide a level of understanding appropriate to an informed citizenry as called for in the 2003 IOM report (1, 8). These degree programs will certainly provide students with an understanding of public health issues that can then be utilized in the workforce or graduate level studies. However, if undergraduate degree prepared individuals are to effectively replace individuals completing an MPH degree and fill entry-level public health practitioner positions, the two professional degree programs offered by American universities, the BPH and BSPH degrees, which provide nearly 47% more content than that found in BA and BS degree programs with public health majors, preliminarily appear to offer a more suitable level of training. As the university-based schools of public health possess the faculty expertise, the time has come for their undergraduate public health programs to produce well-trained practitioners for the public health “workforce” to meet the nation’s needs in 2020 and beyond.

Future studies should be conducted to look in depth at the course content of all public health bachelor’s degree programs in order to determine their specific course content as well as reviewing the actual experiences of recent graduates. Obtaining a better understanding of the students’ preparation for becoming part of the public health workforce will also provide important information for developing effective education programs for public health practitioners.

## Conflict of Interest Statement

The authors declare that the research was conducted in the absence of any commercial or financial relationships that could be construed as a potential conflict of interest.
